# Gas-forming pyogenic liver abscess due to *Klebsiella pneumonia*

**DOI:** 10.1590/0037-8682-0635-2021

**Published:** 2022-02-25

**Authors:** Na Wu, Yong-Sheng Yu, Yi Zhang

**Affiliations:** 1 Shanghai Jiao Tong University Affiliated Sixth People’s Hospital, Department of Infectious Diseases, Shanghai, China.

**FIGURE 1:**
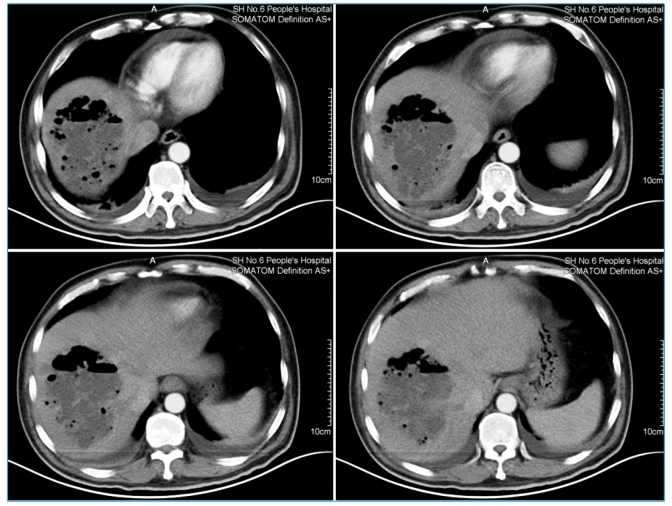
Contrast-enhanced CT scan of the abdomen showing a large abscess (12 cm×11 cm×9 cm) with gas formation in the right lobe of the liver.

A 57-year-old man with poorly controlled diabetes mellitus (DM) was admitted to our hospital with a four-day history of fever, chills, and mild right upper quadrant pain. On physical examination, mild tenderness in the right upper quadrant of the abdomen was observed. Laboratory data showed leukocytosis (15.3×10^9^/L) with hyperglycemia and minor changes in liver values. A contrast-enhanced computed tomography (CT) scan of the abdomen revealed a large gas-forming abscess measuring 12 cm×11 cm×9 cm in the right lobe of the liver (Figure 1). The patient underwent CT-guided percutaneous catheter drainage and was given broad-spectrum intravenous antibiotics and insulin. *Klebsiella pneumoniae* was cultured from both blood and pus. With a gas-forming pyogenic liver abscess (GFPLA) diagnosis, he received six weeks of antibiotic therapy and had a satisfactory response to the medical treatment without any complications.

GFPLA is an uncommon and potentially fatal disease, accounting for 5.6-31.8% of pyogenic liver abscesses^1^. Most patients with GFPLA have poorly controlled DM, which plays an important role in the development of GFPLA^1^. *K. pneumoniae* is the most common pathogen of GFPLA and is found in 85.9% of positive liver pus cultures in GFPLA patients^1^. *K. pneumoniae* infection in DM patients is the cornerstone for the development of GFPLA^2^. The mortality rate of GFPLA ranges from 25.7% to 37.1%^1^. CT findings such as globular configuration, shaggy margins, alveolar internal structure, and total gas content are significant predictors of mortality^3^. To manage GFPLA effectively, appropriate broad-spectrum antibiotics, good control of glucose, and early adequate drainage are compulsory^1^ ^,^ ^2^.
